# Editorial: Structure, Function and Evolution of Complex Cellular Organization in Bacteria and Archaea

**DOI:** 10.3389/fmicb.2021.751416

**Published:** 2021-08-30

**Authors:** Josef D. Franke, John A. Fuerst, Anthony M. Poole

**Affiliations:** ^1^Department of Biology, Creighton University, Omaha, NE, United States; ^2^School of Chemistry and Molecular Biosciences, The University of Queensland, St. Lucia, QLD, Australia; ^3^School of Biological Sciences, University of Auckland, Auckland, New Zealand

**Keywords:** compartmentalization, organelle, nucleus, planctomycete, jumbophage, endospore, phagocytosis, autogenous

New discoveries are transforming our understanding of cell structure and function in Bacteria and Archaea. Whereas previously it has been assumed that complex cell ultrastructure was the exclusive domain of eukaryotes, it is becoming clear that all three domains bear organellar structures, complex intracellular membranes (Grant et al., 2018), and can produce extracellular vesicles (Gill et al., 2019). A recent survey of bacterial cell ultrastructure (Dobro et al., 2017) revealed many diverse, uncharacterized cellular features and structures, suggesting that there is still much to discover. The papers in this Research Topic highlight some of the exciting new developments in our understanding of bacterial and archaeal cell organization, evolution, and architecture.

A central question is whether complex cell architectures in Bacteria and Archaea shed light on the evolutionary origins of eukaryotic cell architecture. On the one hand, mounting evidence for cellular complexity in the newly-discovered Asgard lineages (Zaremba-Niedzwiedzka et al., 2017; Imachi et al., 2020) may help bridge the gap between archaea and the origin of the eukaryotic endomembrane system. However, it is less clear how or whether bacterial ultrastructure relates to eukaryote cellular complexity. One fascinating possibility is that some structures have evolved independently, perhaps converging on similar solutions from very different starting points (Hendrickson and Poole, 2018). A clear example of this is highlighted by the formation of a nucleus-like barrier during jumbophage infection of *Pseudomonas*. This is the subject of a review by Chaikeeratisak et al. The discovery of a “phage nucleus” is exciting in that it indicates there may be multiple circumstances where genetic material is compartmentalized from other parts of a cell. A stunning feature of this structure is that, in contrast to the eukaryote nucleus, this barrier is proteinaceous. The initial, mid cell positioning of the phage nucleus, and its later rotation during new phage assembly, is mediated by a spindle composed of phage-encoded proteins including PhuZ, which is evolutionarily related to tubulin.

Proteinaceous compartments are likely to be a general feature of bacteria, as reviewed by Asija et al., who examine the distribution of bacterial microcompartments (BMCs) based on a survey of the human microbiome. In contrast to jumbophage, which create a shell that sequesters the phage genome to prevent degradation by host-encoded defense mechanisms, some BMCs serve to sequester toxic aldehyde intermediates formed during metabolic reactions that occur within these compartments.

Sequestration of metabolic reactions is also a feature of membrane-bounded bacterial compartments, including anammoxosomes from planctomycetes (Neumann et al., 2014). The phylum Planctomycetes is diverse with a range of complex membrane architectures (Fuerst and Sagulenko, 2011; Wiegand et al., 2020), and Seeger et al. add to this catalog. They present a detailed study on *Tuwongella immobilis*, a relative of *Gemmata obscuriglobus*. *Gemmata* has been the subject of extensive study regarding its complex cell ultrastructure, including debate as to whether it contains a membrane-bounded genetic compartment (Santarella-Mellwig et al., 2013; Sagulenko et al., 2014, 2017; Wiegand et al., 2018). The present work, which made use of FIB-SEM (Focused Ion Beam-Scanning Electron Microscopy) tomography, suggests that *T. immobilis* does not contain such a compartment. Rather, Seeger et al. paint a picture of a complex intracellular membrane, replete with tunnels and caves, which they speculate may create environments where different molecular processes may be spatially separated. The question of whether any bacteria possess nucleus-like compartmentation remains an active area of investigation, with a recent isolate of the candidate phylum Atribacteria containing structures (Katayama et al., 2020) perhaps consistent with genetic compartmentation (Katayama et al., 2020; van Teeseling and Jogler, 2021). In considering the challenges of identifying bacterial genetic compartments, it is helpful to recall that the eukaryote nucleus is a dynamic structure, being disassembled in many species during mitosis (Güttinger et al., 2009). Thus, it will be interesting to understand not only whether there is genetic compartmentation in bacteria, but also whether this is stable or dynamic.

Another underappreciated facet of prokaryote cell biology is the formation of intercellular bridges, enabling cell-cell communication and gene transfer. New work from Sivabalasarma et al. reveals the extent to which this occurs. Using a combination of electron cryotomography (cryoEM) and fluorescent microscopy, they report that the archaeon *Haloferax volcanii* transports a range of macromolecular complexes including ribosomes across these bridges, connecting the cytoplasm of mating cells.

Beskrovnaya et al. review and compare endospore formation in firmicutes with actinobacterial exospore formation. Their work suggests that endospore formation in firmicutes is ancestral, whereas exospore formation likely evolved following actinobacterial diversification.

Endospore formation is noteworthy within the context of compartmentalization for two reasons. First, endospore development involves an asymmetric cell division followed by one cell engulfing the other. This invites parallels with the recent discovery of phagocytosis by the planctomycete, *Candidatus* Uab amorphum (Shiratori et al., 2019). Second, work on the giant firmicute *Epulopiscium* shows that its endospore-like intracellular daughter cells are formed autogenously (Angert and Clements, 2004). This is intriguing given the likely autogenous origins of several organelles, including the eukaryote nucleus. Understanding these separate origins is likely to shed light on the broader process of autogenesis (Hendrickson and Poole, 2018).

Finally, Caetano-Anollés presents an analysis of Gene Ontology terms associated with proteomes and functionomes from the three domains of life. Perhaps in contrast to the connections being made between Asgard lineages and eukaryote cell complexity, his analysis suggests that Archaea possess a small and relatively homogeneous “vocabulary” in comparison with the greater heterogeneity and complexity observed for Bacteria. Moreover, fold family analysis supports other recent suggestions of an ancient evolutionary link between Bacteria and Eukarya (Devos, 2021), perhaps consistent with some shared basal elements needed for the evolution of compartmentation.

We end with a final thought. To understand organellar evolution, it is worth considering what an organelle is. Organelles are often equated with a membrane-bounded compartment, but some of the examples highlighted here do not fit that narrow definition. It is noteworthy that the most conspicuous eukaryotic organelle, the nucleus, is continuous with the cytoplasm and dynamic, being assembled and disassembled during open mitosis. Considering other structures, such as protein-based compartmentation and spatial organization (such as in *Tuwongella* and the nucleolus), will be essential to investigating the origins of complex compartmentation.

## Author Contributions

All authors listed have made a substantial, direct and intellectual contribution to the work, and approved it for publication.

## Conflict of Interest

The authors declare that the research was conducted in the absence of any commercial or financial relationships that could be construed as a potential conflict of interest.

## Publisher's Note

All claims expressed in this article are solely those of the authors and do not necessarily represent those of their affiliated organizations, or those of the publisher, the editors and the reviewers. Any product that may be evaluated in this article, or claim that may be made by its manufacturer, is not guaranteed or endorsed by the publisher.
